# Thoracic spondylolisthesis and spinal cord compression in diffuse idiopathic skeletal hyperostosis: a case report

**DOI:** 10.1186/s13256-017-1252-0

**Published:** 2017-04-01

**Authors:** Yasutaka Takagi, Hiroshi Yamada, Hidehumi Ebara, Hiroyuki Hayashi, Takeshi Iwanaga, Kengo Shimozaki, Yoshiyuki Kitano, Kenji Kagechika, Hiroyuki Tsuchiya

**Affiliations:** 1 Department of Orthopaedic Surgery, Tonami General Hospital, 1-61 Shintomi-cho, Tonami City, Toyama 939-1395 Japan; 2 Department of Rehabilitation Medicine, Kanazawa Medical University, 1-1 Daigaku, Uchinada-machi, Kahoku-gun, Ishikawa 920-0293 Japan; 3 Department of Orthopaedic Surgery, Graduate School of Medicine, Kanazawa University, 13-1 Takara-machi, Kanazawa City, Ishikawa 920-8641 Japan

**Keywords:** Spondylolisthesis, Spinal cord compression, Diffuse idiopathic skeletal hyperostosis, Thoracic spine

## Abstract

**Background:**

Diffuse idiopathic skeletal hyperostosis has long been regarded as a benign asymptomatic clinical entity with an innocuous clinical course. Neurological complications are rare in diffuse idiopathic skeletal hyperostosis. However, if they do occur, the consequences are often significant enough to warrant major neurosurgical intervention. Neurological complications occur when the pathological process of ossification in diffuse idiopathic skeletal hyperostosis extends to other vertebral ligaments, causing ossification of the posterior longitudinal ligaments and/or ossification of the ligamentum flavum. Thoracic spondylolisthesis with spinal cord compression in diffuse idiopathic skeletal hyperostosis has not previously been reported in the literature.

**Case presentation:**

A 78-year-old Japanese man presented with a 6-month history of gait disturbance. A magnetic resonance imaging scan of his cervical and thoracic spine revealed anterior spondylolisthesis and severe cord compression at T3 to T4 and T10 to T11, as well as high signal intensity in a T2-weighted image at T10/11. Computed tomography revealed diffuse idiopathic skeletal hyperostosis at T4 to T10. He underwent partial laminectomy of T10 and posterior fusion of T9 to T12. The postoperative magnetic resonance imaging revealed resolution of the spinal cord compression and an improvement in the high signal intensity on the T2-weighted image.

**Conclusions:**

We report the first case of thoracic spondylolisthesis and spinal cord compression in diffuse idiopathic skeletal hyperostosis. Neurosurgical intervention resulted in a significant improvement of our patient’s neurological symptoms.

## Background

Diffuse idiopathic skeletal hyperostosis (DISH) has long been regarded as a benign asymptomatic clinical entity with an innocuous clinical course [1–3]. DISH rarely causes neurological complications, as evidenced by isolated case reports on the subject; however, if neurological complications do occur, they are often severe enough to warrant major neurosurgical intervention [1–4]. Neurological complications occur in DISH when the pathological process of ossification extends to other vertebral ligaments, causing ossification of the posterior longitudinal ligaments (OPLL) and/or ossification of the ligamentum flavum (OLF) [5]. A retrospective analysis of 74 cases of DISH conducted by Sharma *et al*. found that 11 patients had presented with progressive spinal cord compression or cauda equina syndrome. Of these, OPLL was responsible in nine cases and OLF in two [5]. However, thoracic spondylolisthesis and spinal cord compression in DISH has not previously been reported in the literature. We report the first case of thoracic spondylolisthesis and spinal cord compression in DISH. Neurosurgical intervention relieved the patient’s neurological symptoms significantly.

## Case presentation

**Fig. 1:**
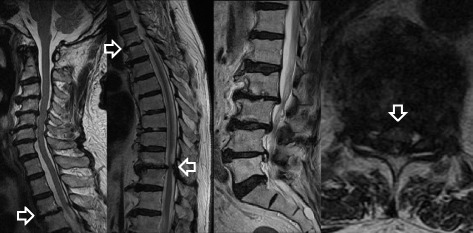
MRI revealed anterior spondylolisthesis and severe cord compression at the T3 to T4 and T10 to T11 levels, as well as high signal intensity on a T2-weighted image at the T10/11 level (*white outline arrows*)

**Fig. 2:**
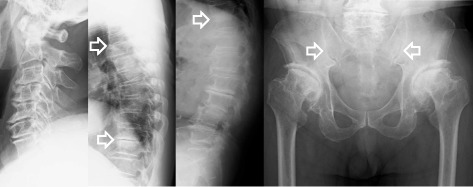
X-ray revealed T3/4 and T10/11 intervertebral disc space narrowing and anterior spondylolisthesis and no involvement of the sacroiliac joints (*white outline arrows*)

**Fig. 3:**
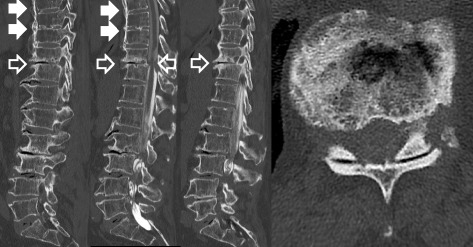
Myelogram-CT revealed T10 to T11 anterior spondylolisthesis and severe cord compression at T10/11 level (*white outline arrows*). Ossification of the posterior longitudinal ligaments and ossification of the ligamentum flavum were not seen at the T10/11 level. DISH was seen above the T10 level (*white solid arrows*)

**Fig. 4:**
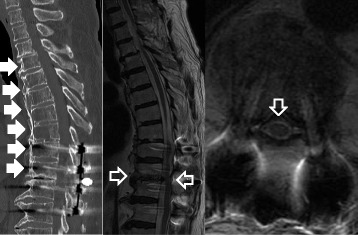
Postoperative CT revealed diffuse idiopathic skeletal hyperostosis between T4 and T10 (*white solid arrows*). Postoperative MRI revealed that spinal cord compression was well decompressed and high signal intensity in T2-weighted image was improved (*white outline arrows*)

A 78-year-old Japanese man presented with a 6-month history of gait disturbance. Magnetic resonance imaging (MRI) of his lumbar spine revealed lumbar spinal stenosis (LSS). He could not walk outdoors. A neurological examination of muscle weakness of his iliopsoas and quadriceps femoris suggested possible spinal cord compression. Cervical and thoracic spine MRI revealed anterior spondylolisthesis and severe cord compression at T3 to T4 and T10 to T11, and high signal intensity on a T2-weighted image at T10/11 (Fig. 1). An X-ray revealed intervertebral disc space narrowing and anterior spondylolisthesis at T3/4 and T10/11 (Fig. 2). A myelogram-computed tomography (CT) scan showed anterior spondylolisthesis and severe cord compression at the T10/11 level. OPLL and OLF were not seen at T10/11. DISH was noted above the T10 level (Fig. 3). We determined that the lesion responsible was located at the T10/11 level. He underwent partial laminectomy at T10 and posterior fusion at T9 to T12. He could walk outdoors with one T-cane postoperatively. Postoperative CT detected DISH between T4 and T10 and anatomical repositioning of the anterior spondylolisthesis previously noted at T10. Postoperative MRI revealed resolution of the spinal cord compression and an improvement in the high signal intensity on the T2-weighted image (Fig. 4).

## Discussion

DISH is a non-inflammatory skeletal disease characterized by calcification and ossification of soft tissues, primarily ligaments and entheses. DISH is also known as senile ankylosing hyperostosis [6]. DISH involving the spine is identified radiologically by flowing ligamentous ossification and calcification of the anterolateral aspect of the vertebral body with relatively well-preserved disc space [7]. The radiographic criteria, as defined by Utsinger *et al.,* includes: (1) bridging osteophytes extending over four contiguous vertebral bodies; (2) relatively normal intervening disk space height in relation to height in relation to age; and (3) absence of apophyseal joints, bony ankyloses, and absence of erosion, sclerosis, or osseous fusion of the sacroiliac joints [8]. Our patient met all these criteria.

Spinal involvement of DISH is characterized radiologically by flowing ossification of the anterior longitudinal ligament, which is typically separated from the anterior aspect of the vertebral body by a thin radiolucent line [9]. The spinal longitudinal ligaments and entheses slowly ossify and show decreased mobility in the affected region until complete ankylosis results. DISH frequently begins in the lower thoracic spinal segments, before extending into the upper thoracic segments and lumbar spine [10].

DISH results in the fusion of several spinal segments, which amplify the biomechanical load on the unaffected segments. Hypermobility of the spinal segment causes disc degeneration or hypertrophy of the OLF, thus resulting in LSS.

The main point of interest in the present case was the slow progression of myelopathy due to T10 anterior spondylolisthesis in a patient with DISH. In this case, DISH between T4 and T10 caused disc degeneration and anterior spondylolisthesis in T3 to T4 and T10 to T11. Hypermobility of the spinal segment gradually led to spinal cord compression.

To the best of our knowledge, this is the first report of thoracic spondylolisthesis and spinal cord compression in a patient with DISH. Neurosurgical intervention provided significant relief of our patient’s symptoms. Patients with significant neurological deficits due to spinal cord compression often require surgical intervention. The type of surgery depends on the site and type of compression.

## Conclusions

We report the first case of thoracic spondylolisthesis and spinal cord compression in DISH. Neurosurgical intervention resulted in significant improvement of our patient’s neurological symptoms.

## Abbreviations

CT: Computed tomography
DISH: Diffuse idiopathic skeletal hyperostosis
LSS: Lumbar spinal stenosis
MRI: Magnetic resonance imaging
OLF: Ossification of the ligamentum flavum
OPLL: Ossification of the posterior longitudinal ligaments
